# Microblogging violent attacks on medical staff in China: a case study of the Longmen County People’s Hospital incident

**DOI:** 10.1186/s12913-017-2301-5

**Published:** 2017-05-19

**Authors:** Jia Tian, Li Du

**Affiliations:** 1 The Office of Nosocomial Infection Control, Zhongnan Hospital of Wuhan University, Donghu Road 169, Wuhan, China; 2 Faculty of Law, University of Macau, E32, Avenida da Universidade, Taipa, Macau SAR China

**Keywords:** Social network, Hospital violence, Weibo, Healthcare crisis

## Abstract

**Background:**

A violent attack on medical staff in Guangdong Province, in which a female doctor at Longmen County People’s Hospital (LCPH) was severely injured by a knife-wielding patient, has drawn significant public attention to the phenomenon of hospital violence and initiated discussions on how to resolve violence in hospitals. Social networking sites, such as Sina Weibo, a Chinese version of Twitter, have played a role in this public debate. The incident at LCPH provides an opportunity to examine how Weibo has been used in the debate about violence against medical staff in China.

**Methods:**

Using the Sina Weibo’s built-in search tool, we established a dataset of 661 Chinese-language micro-blogs containing the search terms: Longmen (“龙门”), doctor (“医生”), and slash (“砍”) that were posted between July 15, 2015, the date of the violent incident at LCPH, and August 15, 2015. We performed a content analysis of the micro-blogs to examine: users’ demographics, attitudes toward the injured doctor and the attacker, possible reasons for the hospital violence, and proposed measures for preventing doctors from violent incidents.

**Results:**

73.2% of the micro-blogs were sent by individual Weibo users, and 26.8% were posted by organizations. For individual users, around 10.0% described themselves as either doctors or healthcare providers, but users from the legal profession were rarely identified. Moreover, only 3 micro-blogs proposed concrete strategies for preventing hospital violence, and nearly 10.0% of micro-blogs expressed regrets about entering a medical career and attempts to quit medical positions. In general 56.3% of micro-blogs showed sympathy for the injured doctor, while less than 25.0% of micro-blogs explicitly condemned the attacker’s behavior.

**Conclusions:**

Weibo users played a role in distributing news information about the violent incident at LCPH; however, the legal perspective is inadequately discussed in the debate, and discussion of constructive measures for protecting doctors and preventing hospital violence was rare. Our research suggests that critical challenges for the Chinese health care system will remain or become worse if no effective measures are implemented to prevent hospital violence.

## Background

On July 15, 2015, a collection of photos of a bloody human hand and sliced shoulders made headlines throughout Chinese news media websites. The victim is Dr. Ou Lizhi, a female physician at Longmen County People’s Hospital (LCPH) in Guangdong Province. On July 15, she was making the morning rounds while a man named Liao approached her. The man said he was her patient a year ago and was not feeling good. He asked Dr. Ou for medical attention, but she told him that he had to wait until she finished checking the wards. Liao suddenly pulled out a kitchen knife from his bag and swung it at the doctor. Dr. Ou dodged the knife but, sadly, did not escape injury. The bones in her right hand were fractured and she received deep cuts in her shoulders [1]. Due to her critical condition, she was transferred to a higher-level hospital for treatment and the operation alone took more than 10 hours [1]. According to her surgeon’s preliminary inspection, she might not regain full use of the injured hand.^1^ The tragedy quickly drew public attention to the condition of Dr. Ou and discussions around the phenomenon of hospital violence [2].

Unfortunately, Dr. Ou’s case is not rare in China. With the deteriorating patient-physician relationship, the number of violent attacks in hospitals has increased over the past couple of years [3, 4]. Violent incidents often occur after medical malpractice or even when treatment results do not meet patients’ expectations [5]. In most cases, patients and their relatives use verbal abuse, or threats against physicians to express their dissatisfaction and disappointment with the treatment results. In some extreme cases, such as Dr. Ou’s incident, physical violence results in injury, death, and psychological harm [5, 6]. A 2012 article in *Time* magazine raised global awareness of the issue of hospital violence in China [7], and in 2014, an editorial in the *Lancet* indicated that a third of China’s doctors had experienced conflict and thousands had been injured in violent incidents [8]. A recent 2015 physician practice report issued by the Chinese Medical Doctor Association indicated that, from 2009 to April 2015, there were more than 105 incidents resulting in severe injury in China [9].

Sina Weibo, or micro-blogs, a Chinese version of Twitter, is a popular social media platform in China. With more than 200 million active users, Weibo has become an influential forum for spreading news information and engaging in public debate [10]. There is a growing body of literature that suggests that Weibo can have a significant impact on the mobilization of online public opinion and the promotion of mutual understanding in the context of health–related social issues [11]. With participation of users from various backgrounds, Weibo can reflect and shape public perceptions and public discourse [12]. In the context of hospital violence, there has been little research on the role, tone and nature of social media, such as who has interest in spreading the news and is engaged in discussions, what content has been shared on Weibo, and how Weibo is being used to promote the protection of doctors and prevention of hospital violence. The LCPH incident creates an opportunity for an exploration of these issues. In this paper, we explore how perspectives on the LCPH incident and related opinions on stopping hospital violence are presented on the Weibo, or micro-blogs. We also relate the findings about Weibo users’ perspectives to the impact of the endless hospital violence in the Chinese healthcare system.

## Methods

We used the Sina Weibo’s built-in search tool to collect data and develop a data set. To ensure that micro-blogs relating to Longmen hospital violence can be fully extracted, we conducted an exploratory search on the Sina Weibo to determine a list of relevant search terms. As a result of the exploratory search, three Chinese search terms were identified, and they included: Longmen (“龙门”), doctor (“医生”), and slash (“砍”). We limited the search period to the dates between July 15, the date of the story was first reported, and August 15, 2015. This generated a corpus of data consisting of 664 Chinese original micro-blogs, that is, we did not include micro-blogs that had been re-posted by other users. Three micro-blogs were excluded from the dataset, because they did not specifically discuss the LCPH incident. 661 micro-blogs constitute the final dataset for this study.

The content analysis was conducted in two stages. We first conducted an exploratory thematic analysis on 10% of the dataset. Based on these findings, we developed a coding framework that include the following twelve questions: 1) Is the sender an organization or individual user? 2) If it is posted by an organization, what type of organization is it? 3) If it is sent by an individual user, what is his/her background? 4) Does the text show sympathy to the injured doctor? 5) Does the text condemn the attacker’s behavior? 6) Does the text mention that the hospital’s poor medical treatment is one of the reasons for the tragedy? 7) Does the text express disappointments with the government? 8) Does the micro-blog indicate that the deteriorating patient-physician relationship is the reason for the tragedy? 9) Does the text mention the attempt to quit the doctor’s job or express regret about studying medicine? 10) Does the text mention that measures should be developed to protect doctors from being injured? 11) Does the text include a statement of “stop violence?” and 12) Does the text mention that the hospital should disclose the incident publicly?

One of the authors coded the entire dataset. Given the subjective nature of the content analysis, an independent coder also coded approximately 10% of the micro-blogs in our dataset (*n* = 66). We used Cohen’ Kappa to calculate the inter-coder agreement. Initial calculation of Kappa scores revealed poor agreement for two items, question 4 and 5. To resolve disagreement, the two coders held a meeting and arrived at a consensus on the definition of “sympathy” and “condemnation.” After recoding based on the agreed upon definitions, Kappa scores ranged from 7.13 to 1.00, indicating substantial to perfect agreement [13].

## Results

The majority (73.2%) of micro-blogs (*n* = 484) were published by individual Weibo users, and the remaining micro-blogs (*n* = 177) were posted by organizations (26.8%). Among the 484 individual users, 48 described themselves as either doctors or health care providers, while users from the legal profession were rarely identified. With regard to the organizations, news media and medical institutions were the two major micro-blog publishers, who posted 97 and 24 micro-blogs respectively. 3 micro-blogs were published by courts and legal service agencies, i.e., the China’s Supreme People’s Procuratorate, Heze Yuye County Court, and Democracy and Legal System Official Website.

Seven micro-blogs indicated that the deteriorating patient-physician relationship was the cause of the problem. 20 micro-blogs expressed disappointment with the Chinese government, arguing that poor protection of physicians in China is the fault of the government. Moreover, 33 micro-blogs were very dissatisfied with the LCPH’s silence after the incident. They urged that the head of the hospital to speak up, disclose the facts of the incident, and publicly condemn the attacker’s violent behavior (see Table 1).

**Table 1:** Examples of micro-blogs^a^

Senders	Micro-blog texts
Organizations	
人民日报 People’s Daily	【广东惠州一女医生被患者连砍数刀 肌腱断裂血肉模糊】欧丽志从医14年,广东龙门县人民医院医生。前日查房时,一男患者廖某找到她,她称要等查完房、开完医嘱再帮他看病。男子突然掏出一把菜刀,连砍她数刀。她的肩、手臂、腿都被砍伤,刀口很深,血肉模糊。目前廖某已被刑拘。 【A woman doctor in Huizhou Guangdong was slashed by a patient, rupture of tendon badly mutilated】Ou Zhili been practiced medicine for 14 years is the doctor at the People’s Hospital in Longmen County Guangdong. Ou was making her morning rounds when a male patient Liao approached her. She said he had to wait until she finished checking the wards. The man suddenly pulled out a kitchen knife and raised it at Ou slashing her on the arms, legs and shoulder, deep wounds, badly mutilated. Liao had been detained.
南方都市报 Southern Metropolis Daily	【惠州一女医生被患者连砍数刀】欧丽志,广东惠州龙门县人民医院医生,从医14年。前日查房时,一男患者廖某找到她,希望她帮他看病,她称要等查完房开完医嘱再帮他看,男子连砍她数刀。送医路上,她一直在哭。仅手术就用了10小时。"发生这样的事,谁还愿意做医生?"廖某已被刑拘。 【Huizhou a woman doctor was slashed several times by a patient】Ou Lizhi, a Guangdong Huizhou Longmen County People's Hospital doctor, been practiced for 14 years. The day before yesterday, when she was making rounds, a male patient Liao found her and hoped her to see his illness, she said he had to wait until she finished checking the wards, the man slashed her several times. On the way to the hospital, she had been crying. Operation alone took 10 hours. “Things like this happen, who would like to be a doctor?” Liao had been detained.
医学界网站 Medical website	【惠州市龙门县人民医院一名内科女医生被患者砍伤】7月15日,一位34岁男子廖某将刀藏在包内带进龙门县人民医院,接诊的主任医生说等会过来打针,廖某没来由就挥刀连砍女医生,“手都差点掉了,地上都是血,身上也有几刀”。目前这名女医生因伤情较重送往广州中山一院救治。哀莫大于心死…… 【Huizhou City Longmen County People’s Hospital a woman physician was slashed by a patient】on July 15, a man named Liao 34-year-old hidden a knife in a bag and walked in Longmen County People’s Hospital, the chief physician said the injection would be done in a while, Liao slashed the woman physician gratuitously. “Hands almost fell on the ground, blood’s everywhere, and there were blades on the body.” Currently the woman physician was sent to the Guangzhou Zhongshan First Hospital for treatments because of the severe injuries. Nothing is more lamentable than a dead heart…
佛山日报 Foshan Daily	【广东省:各医疗机构设置警务室】7月15日,惠州龙门县人民医院神经内科欧丽志医生在医院病房查房时,遭到前来就医的患者砍成重伤。昨晚,广东省卫计委发出紧急通知,要求各医疗机构设立警务室或(和)警车,安装视频监控设备和报警装置,建立中心监控室,24小时全方位、实时监控。 【Guangdong Province: Medical institutions establish police rooms 】July 15, 2015, Dr. Ou Zhili a neurologist at Guangdong Longmen County People’s Hospital was slashed by a patient to severe injury when she was making her rounds. Last night, Guangdong Provincial Health and Family Planning Commission issued an emergency notice, requiring each medical institution establish a police room or (and) patrol wagons, install video surveillance equipment and alarm devices, and establish a central monitoring room to implement 24-hour real-time supervisions.
Individuals^b^	
医### Medical###	【痛】7月15日上午9时,广东惠州龙门县人民医院神内科欧医生正在查房,一位34岁的门诊男患者廖某(非住院)来到病房,说一年前曾经找她看过病,现在又不舒服了,要求欧医生必须帮他看病。欧医生说等查完房再帮他看,结果患者一怒之下突然从怀里拿出菜刀连砍猝不及防的医生。 【SAD】At 9:00 on July 15, People's Hospital Longmen County, Huizhou, Guangdong, Dr. Ou a neurologist was making her morning rounds, a 34 -year-old male patient Liao (out-patient) came to the ward, saying that a year ago she had once seen his condition and now he’s uncomfortable again. He asked the doctor to check his sickness. Dr. Ou said she would treat him until she finished checking the wards. As a result, the patient suddenly pulled out a kitchen knife and slashed the doctor who was off her guard.
乳### Breast###	转来自朋友圈:今天,下乡坐诊,就在我的身边,广东龙门县一内科医生无辜被一暴徒砍至重伤!她还很年轻,才38岁,一名很善良的好医生,在没有一点征兆的情况下被袭击,完全没有躲避的机会!就因为她是一名医生,就因为她在上班,她下半辈子的幸福就这样被毁了!而我们,只能为她祈祷!@健康中国 Forwarded from a friend’s moment: today, went to the countryside and provided medical services, just beside me, an innocent doctor in Longmen County hospital was slashed by a mob, severely injured! She is young, only 38 year-old, a very kind good doctor, in the absence of signs that the attack happened, no chance to escape! Just because she is a doctor, she is on duty; the rest of her life was destroyed! And we, can only pray for her! @ HealthChina
健### Health###	强烈谴责!@宁大附院王伯军 @急救医生贾大成 @手术刀_李医生 @千里马常有@徐毓才 @友悦//@华南医疗圈: //@赖清辉:广东龙门县一内科女医生被砍重伤!//@万爱哥://@医事律师李惠娟: //@乳腺科医生:身边的杯具越来越多!//@北界松:我一个朋友的同学 //@病理医生杨连君://@乳腺科医生: 别点赞了。 Strongly condemn! @宁大附院王伯军 @急救医生贾大成 @手术刀_李医生 @千里马常有@徐毓才 @友悦//@华南医疗圈: //@赖清辉: Guangdong Longmen County a woman physician was slashed to severe injury //@万爱哥://@医事律师李惠娟: //@乳腺科医生: tragedies around are increasing! //@北界松: a schoolmate of my friend //@病理医生杨连君://@乳腺科医生: don’t click “like.”
手### Hand###	单纯的谴责已毫无意义了,医护们应该想想如何转行远离暴力,因为我们已经承受不起了。 //@健康使者李宇森:强烈谴责!@宁大附院王伯军 @急救医生贾大成 @手术刀_李医生 @千里马常有@徐毓才 @友悦 //@华南医疗圈: //@赖清辉:广东龙门县一内科女医生被砍重伤!//@万爱哥://@医事律师李惠娟: Condemnations are meaningless. Medical workers shall think about how to switch to another job to keep away from violence, cause we cannot afford to it. //@健康使者李宇森: strongly condemned!@宁大附院王伯军 @急救医生贾大成 @手术刀_李医生 @千里马常有@徐毓才 @友悦 //@华南医疗圈: //@赖清辉: A female physician in Guangdong Longmen County was slashed to severe injury !//@万爱哥://@医事律师李惠娟:
R###	砍的好!一名医生在医院被砍成这样都没有医护和医院官方正式公开发声,没有医护和官方举办新闻发布会公布事情经过澄清事实真相,试想如果是一个患者在医院被砍成这样或者在医院被盗肾,可能会有医护医院站出来实话实说吗?龙门县人民医院伍耀堂院长,你站出来!我保证不打死你。 Well done! A doctor was slashed so badly, but no healthcare providers’ and the hospital’s official voice, no healthcare providers and officials made a press conference to announce and clarify the truth of the incident, imagining that if a patient is slashed in a hospital or a patient’s kidney is stolen in a hospital, will a healthcare provider or hospital official stand out to tell the truth? The Dean of Longmen County People’s Hospital, Wu Yaotang, you stand up! I promise not to beat you to death.

^a^Translated by the authors

^b^Authors anonymized the users’ Weibo names for privacy concerns

15.9% of the micro-blogs (*n* = 105) mentioned that measures should be taken to improve the protection of health care providers’ personal safety and prevention of hospital violence. Slogans such as “stop violence,” or “no violence” were advocated by 15 micro-blogs. Nonetheless, with the exception of 3 micro-blogs that proposed concrete measures to stop hospital violence, the remaining micro-blogs did not suggest any constructive strategies. At the same time, 8.3% of micro-blogs (*n* = 55) expressed regrets about studying medicine or entering a medical career and attempts to quit their doctor’s positions. Among these micro-blogs, 8 were sent by Weibo users who identified themselves as healthcare providers.

Our data indicates that 56.3% of micro-blogs (*n* = 372) showed sympathy for the injured doctor. They used emotional adjectives, i.e., “sad,” “miserable,” “pray,” “crying,” and “horrible” to express their shared feelings (see Table 1). 43.4% of micro-blogs (*n* = 287) did not contain any emotional expressions in regards to the incident. 22.5% of micro-blogs (*n* = 149) explicitly condemned the attacker’s violent behavior. Moreover, 3 micro-blogs recalled their experience at LCPH, criticizing the hospital’s poor medical conditions and doctors’ irresponsible attitude. Two other micro-blogs, by blaming the doctors’ bad medical performance and attitude in general, even supported to the patient’s attack.

## Discussion

Our findings indicate that news media organizations, both the party-owned and market-oriented news media institutions, actively spread information about the violent incident at LCPH using Weibo. However, news media organizations are not the only sources of information. Other Weibo users such as medical institutes and individual micro-bloggers also played a role in spreading information. This finding is in accordance with existing social networking research that has found that news media organizations play a role, though not a dominating one, in disseminating information in the era of social networking [14]. Our analysis also finds that individual users not only promoted the spread of the information, but also actively engaged in discussions and debates.

Given that doctors are the victims of the hospital violence, it is understandable that many doctors used Weibo actively to spread the news and engage in discussions. However, it is worth noting that individual users from the legal profession were absent from discussions. In the context of hospital violence, numerous legal issues associated with healthcare, e.g., patient-physician relationships and physicians’ rights, need to be carefully and promptly addressed, and lawyers and legal scholars should play a key role in leading and shaping the discussions. Nonetheless, legal discourses on how to prevent violence against physicians and compensation for the injured doctor were rarely mentioned. The lack of representation of and attention paid from the legal profession renders the discussion of hospital violence incomplete, i.e., the debate is missing the rule of law perspective, and, furthermore, contributes to deficient legal protection of doctors and the healthcare system in general [15]. According to the judgment made by the Longmen County People’s Court on March 30, 2016, the attacker was sentenced to two years imprisonment [16].

Many micro-blogs claimed that actions should be taken to stop hospital violence and improve the protection of healthcare providers’ life rights. However, with the exception of micro-blogs originally posted by Foshan Daily (see Table 1), the majority of micro-blogs did not include any constructive suggestions or recommendations. This may reveal some real challenges that the Chinese government is facing, i.e., measures to stop the hospital violence have been ineffective thus far [17]. A series of severe hospital attacks that occurred in May 2016, including an incident in Guangdong province, in which Dr. Zhongwei Chen was brutally killed by a patient, indicates that the deteriorating doctor-patient relationship has not improved significantly, and that no effectual methods have yet been developed to stop the violence.

Without effective measures, the attacks against physicians become unpreventable and endless. Less than 25.0% of micro-blogs that explicitly condemned the attacker’s behavior reveal possible permissive attitudes of Weibo users towards the phenomenon of hospital violence. One micro-blog argued: “condemnation is not meaningful anymore; doctors should escape from this chaos and switch careers” (see Table 1). Indeed, we found that nearly 10.0% of micro-blogs expressed regrets about studying medicine or entering a medical career and attempts to quit doctor positions. Recent news reports and surveys suggest that such career changes are in fact taking place [4, 5, 18]. The decreasing number of doctors will aggravate the shortage of physicians, and this will bring critical challenges to the current Chinese health care system.

A long-term impact of the hospital violence on the healthcare industry would be far-reaching. Existing surveys have demonstrated that the anxiety and insecurity surrounding careers in medicine have inhibited talented students, new blood, from entering medical schools [19]. If the failure to attract elite students into the medical community persists, it will set back Chinese medical research and the quality of healthcare in the future. Accordingly, in the face of the already large and aging population, along with a shortage of physicians and low quality healthcare, a health care crisis is foreseeable. What’s more challenging is that this health crisis will not be limited to China. As indicated by a recent “fake” vaccine crisis in China and the loss of confidence in the Chinese vaccination system, Chinese patients might rush to health care centers abroad, triggering issues in the local health care systems of nearby countries and regions [20].

## Conclusions

In summary, social media can play a role in reflecting and shaping public opinion and policy decisions [12] and our findings demonstrate that Weibo users played a role in distributing information about the violent incident at LCPH, and more than half of the micro-blogs showed sympathy for the injured doctor. However, the legal perspective was inadequately represented in the debate, and the exchange of views on constructive measures for protecting doctors and preventing hospital violence was rare. Our research indicates that some Weibo users consider doctors to be a vulnerable population and that more effective measures are required to prevent hospital violence. However, without the presence of valuable legal perspectives in these public debates, the threats to the safety of Chinese healthcare providers may remain or become worse, and perhaps the growing rate of hospital violence may even trigger crises in the domestic health care system.

## Abbreviation

LCPH: Longmen County People’s Hospital

## References

[1] South China Morning Post. Knife-wielding man attacks Chinese woman doctor making her rounds in hospital. 2015. http://www.scmp.com/news/china/society/article/1840836/knife-wielding-man-attacks-chinese-woman-doctor-while-making-her. Accessed 15 May 2017.

[2] China Daily Europe. 600,000 Chinese doctors sign petition against hospital violence. 2015. http://europe.chinadaily.com.cn/china/2015-07/19/content_21326515.htm. Accessed 15 May 2017.

[3] Hesketh T, Wu D, Mao LN, Ma N (2013). Violence against doctors in China. BMJ.

[4] Pan Y, Yang XH, Jiang PH, Tan HG, Xiao LZ, Hui FG (2015). To be or not to be a doctor, that is the question: a review of serious incidents of violence against doctors in China from 2003-2013. J Public Health.

[5] Xing K, Jiao ML, Ma HK, Qiao H, Hao YH, Li Y (2015). Physical violence against general practitioners and nurses in Chinese township hospitals: a cross-sectional survey. PLoS ONE.

[6] Tu J. Yinao: protest and violence in China’s medical sector. 2014. http://berkeleyjournal.org/2014/12/yinao-protest-and-violence-in-chinas-medical-sector/. Accessed 15 May 2017.

[7] Rauhala E. Why China’s doctors are getting beat up. 2014. http://time.com/15185/chinas-doctors-overworked-underpaid-attacked/. Accessed 15 May 2017.

[8] The Lancet (2014). Violence against doctors: Why China? Why now? What next?. Lancet.

[9] Chinese Medical Doctor Association. Chinese Physician Practice White Paper 2015. 2015. http://www.cmda.net/xiehuixiangmu/falvshiwubu/tongzhigonggao/2015-05-28/14587.html. Accessed 15 May 2017.

[10] Chang XY. China’s Weibo political and social implications?. Education About ASIA. 2013;18(2):16–20.

[11] Fung ICH, Hao Y, Cai JX, Ying YC, Schaible BJ, Yu CMJ (2015). Chinese social media reaction to information about 42 notifiable infectious diseases. PLoS ONE.

[12] Guan WQ, Gao HY, Yang MM, Li Y, Ma HX, Qian WN, et al. Analyzing user behavior of the micro-blogging website Sina Weibo during hot social events. Physica A. 2014. doi:10.1016/j.physa.2013.09.059.

[13] Landis JR, Koch GG (1977). The measurement of observer agreement for categorical data. Biometrics.

[14] Wu YF, Atkin D, Lau TY, Lin C, Mou Y (2013). Agenda setting and micro-blog use: an analysis of the relationship between Sina Weibo and newspaper agendas in China. J Soc Media Soc.

[15] Tucker JD, Cheng Y, Wong B, Gong N, Nie JB, Zhu W (2015). Patient-physician mistrust and violence against physicians in Guangdong Province, China: a qualitative study. BMJ Open.

[16] Lu SY, Huida Q. The man who slashed the doctor at Longmen County People’s Hospital sentenced 2-year imprisonment. 2016. http://news.medlive.cn/all/info-news/show-93635_97.html. Accessed 15 May 2017.

[17] Yao SK, Zeng Q, Peng MQ, Ren SY, Chen G, Wang JJ (2014). Stop violence against medical workers in China. J Thorac Dis.

[18] Xing M. The rise of patient-on-doctor violence in China. 2016. http://bowdoinglobalist.com/2016/04/15/the-rise-of-patient-on-doctor-violence-in-china/. Accessed 15 May 2017.

[19] Jie L (2012). New generations of Chinese doctors face crisis. Lancet.

[20] Li S, Chui T. Vaccine scare sends mainland parents to HK. 2016. http://www.chinadailyasia.com/hknews/2016-03/29/content_15407638.html. Accessed 15 May 2017.

